# Biochemical characterization of an acetylesterase from *Bacillus subtilis* and its application for 7-aminocephalosporanic acid deacetylation

**DOI:** 10.3389/fmicb.2023.1164815

**Published:** 2023-05-03

**Authors:** Xiaoliang Wang, Sujin Nong, Jiayi Li, Yan Liu, Qian Wu, Zunxi Huang, Bo Xu, Junmei Ding

**Affiliations:** Engineering Research Center of Sustainable Development and Utilization of Biomass Energy, Ministry of Education, Yunnan Normal University, Kunming, China

**Keywords:** 7-ACA, D-7-ACA, deacetylation, esterase, acetylesterase

## Abstract

Deacetyl-7-aminocephalosporanic acid (D-7-ACA), which could be converted from 7-aminocephalosporanic acid (7-ACA), is a crucial starting material that is used for synthesizing industrial semisynthetic β-lactam antibiotics. Enzymes involved in the conversion from 7-ACA to D-7-ACA present critical resources in the pharmaceutical industry. In the present study, a putative acetylesterase, EstSJ, identified from *Bacillus subtilis* KATMIRA1933, was first heterologously expressed in *Escherichia coli* BL21(DE3) cells and biochemically characterized. EstSJ belongs to carbohydrate esterase family 12 and is active on short-chain acyl esters from *p*-NPC_2_ to *p*-NPC_6_. Multiple sequence alignments showed that EstSJ was also an SGNH family esterase with a typical GDS(X) motif at its N-terminal end and a catalytic triad composed of Ser^186^-Asp^354^-His^357^. The purified EstSJ displayed the highest specific activity of 1,783.52 U mg^–1^ at 30°C and pH 8.0, and was stable within the pH range of 5.0–11.0. EstSJ can deacetylate the C3′ acetyl group of 7-ACA to generate D-7-ACA, and the deacetylation activity was 4.50 U mg^–1^. Based on the structural and molecular docking with 7-ACA, the catalytic active sites (Ser^186^-Asp^354^-His^357^) together with four substrate-binding residues (Asn^259^, Arg^295^, Thr^355^, and Leu^356^) of EstSJ are revealed. This study provided a promising 7-ACA deacetylase candidate that could be applied to produce D-7-ACA from 7-ACA in the pharmaceutical industry.

## Introduction

Cephalosporins are a class of β-lactam antibiotics that are widely used to treat and prevent diseases caused by bacteria that disrupt the synthesis of the peptidoglycan layer of the bacterial cell wall (Guo et al., 2016). However, due to uncontrolled use and a continuous production of cephalosporins, an increasing number of pathogenic bacteria with antibiotic-resistance genes that can escape from various types of antibiotics have emerged. Therefore, exploring and developing new types of effective antibacterial antibiotics are urgent (Theuretzbacher et al., 2020). Deacetyl-7-aminocephalosporanic acid (D-7-ACA) is an important intermediate for synthesizing new kinds of semisynthetic cephalosporins due to the modification of the 3′ hydroxyl group of D-7-ACA which is easy to be achieved (Tan et al., 2018), such as the generation of clinically used cefoxitin and cefuroxime (Ma et al., 2015, 2016). Two major methods, namely, chemical and enzymatic deacetylation of 7-ACA, could be used to generate D-7-ACA. The enzymatic processes using deacetylases or esterases have attracted more attention and have gradually become the prevailing trend for the production of D-7-ACA, compared with the chemical approaches that require energy-intensive conditions and generate toxic waste (Ma et al., 2016).

Esterases (EC 3.1.1.X) that can hydrolyze esters or synthesize pure compounds are widely used for the preparation of commercial products that are used in the biotechnological and pharmaceutical industries (Zhang et al., 2017). Esterases or deacetylases are typically classified into 20 carbohydrate esterase (CE) families in the Carbohydrate-Active Enzymes (CAZy) database^1^ based on their activities on carbohydrate ligands and deacetylases or esterases from the CE7 or the CE12 family exhibiting deacetylation activity toward cephalosporins are included. To date, several esterases, namely, Axe1 (*Thermoanaerobacterium* sp. JW/SL-YS485) (Lorenz and Wiegel, 1997), Axe (*Bacillus pumilus* PS213) (Degrassi et al., 2000), CAH (*Bacillus subtilis* 168) (Vincent et al., 2003), TM0077 (*Thermotoga maritima* MSB8) (Levisson et al., 2012), AXE (*Bacillus subtilis* CICC 20034) (Tian et al., 2014), PbAcE (*Paenibacillus* sp. R4) (Park et al., 2018), and EstZY (*Alicyclobacillus tengchongensis*) (Ding et al., 2020) from the CE7 family have been characterized and demonstrated their activities on 7-ACA or cephalosporin C. Additionally, among the CE12 family, YesT from *Bacillus subtilis* ATCC 6633 (Martínez-Martínez et al., 2008), BH1115 from *Bacillus halodurans* C-125 (Navarro-Fernández et al., 2008), and EstD1 from *A. tengchongensis* (Ding et al., 2016) also exhibited 7-ACA deacetylation activity. Although deacetylases or esterases that are active on 7-ACA have good industrial and pharmaceutical potential, reports on such enzymes, especially with high deacetylation activities, are still limited.

Esterases belonging to α/β superfamily enzymes contain a catalytic triad (S-D/E-H) and a conserved pentapeptide motif (GXSXG) near the nucleophilic serine residue (Bhatt et al., 2021). Conversely, the SGNH hydrolase family does not contain a classical GXSXG motif and is characterized by the presence of a highly conserved, four-residue sequence motif near the N-terminal end (Ser-Gly-Asn-His, SGNH) (Kim et al., 2017; Maršavelski et al., 2020). The SGNH hydrolases display broad substrate specificities and could be applied in various fields (Maršavelski et al., 2020). For example, LpSGNH1 (PDB ID: 3DC7), an SGNH esterase from *Lactobacillus plantarum*, was active on *p*-nitrophenyl acetate, acetyl xylan, glucose pentaacetate, cefotaxime, and 7-ACA (Kim et al., 2017). The catalytic mechanisms of SGNH hydrolases were also diverse, such as MsAcT (PDB ID: 2Q0S) from *Mycobacterium smegmatis* exhibiting both transesterification and side reaction hydrolysis activities (Kazemi et al., 2018). However, besides BH1115 (Navarro-Fernández et al., 2008) and EstD1 (Ding et al., 2016), limited information about the characterization of SGNH hydrolases with 7-ACA deacetylation activities from microorganisms is available.

In this study, an acetylesterase, EstSJ, identified from probiotic bacteria *Bacillus subtilis* KATMIRA1933 (Karlyshev et al., 2014) and their biochemical characteristics were evaluated in detail. The sequence similarity, substrate binding, and activity on 7-ACA were determined through sequence alignment and kinetic analyses.

## Materials and methods

### Materials and chemicals

*p*-nitrophenol (*p*-NP) acetate (C_2_), butyrate (C_4_), caproate (C_6_), cefotaxime acid, tert-butyl acetate, 7-ACA, glyceryl tributyrate, cephalothin sodium, glyceryl trioleate, cefuroxime acid, and terpinyl and linalyl acetates were obtained from Sigma-Aldrich (Merck, Germany) or TCI (Tokyo, Japan). Qiagen (Hilden, Germany) provided Ni–NTA agarose. The K-ACET kit was obtained from Megazyme (Dublin, Ireland). The *Fast* Mutagenesis System, pACYCDuet-1 plasmid, and One-Step Cloning Kit were obtained from TransGen Biotech (Beijing, China), Miaoling Bioscience and Technology (Wuhan, China), and Vazyme Biotech (Nanjing, China), respectively. Synthesis of primers and *estSJ* gene was conducted by Sangon Biotech (Shanghai, China).

### Sequencing analysis, cloning, and expression of EstSJ

The nucleotide and protein sequences of a putative acetylesterase, EstSJ (Locus tag: JMEF01000001.1:136417-137565; Protein ID: KDE25464.1), were revealed in the genomic sequence of *B. subtilis* KATMIRA1933 (GenBank Accession Number: JMEF00000000.1). The signal sequence of EstSJ was predicted using SignalP 4.0 (Petersen et al., 2011). Phylogenetic analysis of EstSJ with other homologs was conducted by MEGA 7.0 (Kumar et al., 2016). CAZy database was applied to classify EstSJ. Then, the *estSJ* gene was commercially synthesized and ligated into the pACYCDuet-1 vector by *Bam*HI and *Not*I restriction sites with in-frame 6 × His tag sequence fused at the C-terminus. EstSJ mutants, including the substitutions of potential catalytic triads (Ser^186A^, Asp^354A^, and His^357A^) and other residues (Asn^259A^, Arg^295A^, Thr^355A^, and Leu^356A^), were constructed by site-directed mutagenesis (where A represents alanine). Primers used are shown in Supplementary Table 1, and the correct inserts were confirmed through DNA sequencing.

The recombinant plasmids, namely, wild-type pACYCDuet-1/EstSJ and its mutants pACYCDuet-1/EstSJ^Ser186A^ (EstSJ^Asp354A^, EstSJ^His357A^, EstSJ^Asn259A^, EstSJ^Arg295A^, EstSJ^Thr355A^, and EstSJ^Leu356A^), were transformed into *Escherichia coli* BL21(DE3) competent cells to heterologously overexpress EstSJ and its mutants, respectively. The recombinant *E. coli* BL21(DE3) cells were cultivated in Luria–Bertani (LB) medium supplemented with 12.5 μg mL^–1^ of chloramphenicol at 37°C until OD_600_ reached 0.5–0.7. Then, the final concentration of 0.7 mM isopropyl β-D-1-thiogalactopyranoside (IPTG) was added and the recombinant cells were continuously cultivated for another 20 h for the induction of EstSJ and its mutants. To avoid the formation of inactive inclusion bodies during overexpression, the cultivation temperature of induced recombinant cells was shifted from 37°C to 16°C (Gileadi, 2017).

### Purification of EstSJ and its variants

To purify EstSJ and its variants, the recombinant cells were harvested by centrifugation (8,000 × *g*, 20 min, 4°C) and ultrasonically disrupted (7 s, 150 w) in a binding buffer containing 20 mM Tris–HCl, 10% glycerol, and 500 mM NaCl, pH 8.0, for 15 min on ice. The supernatant was collected by centrifugation (12,000 × *g*, 30 min, 4°C) and purified by a Ni–NTA agarose column. The recombinant EstSJ/mutants were washed with a binding buffer containing 8 mM imidazole and eluted with a linear imidazole gradient (20-500 mM) in the binding buffer. The purity and concentration of EstSJ were determined by sodium dodecyl sulfate-polyacrylamide gel electrophoresis (SDS-PAGE) and Bradford method (Bradford, 1976).

### Enzyme assay

The standard esterase assay buffer containing 0.6 mM *p*-NPC_2_ and 1.12 mg mL^–1^ EstSJ in 50 mM Tris–HCl buffer, pH 8.0, and the reaction mixture was incubated at 30°C for 5 min (Levisson et al., 2007). Subsequently, the reaction was terminated by the addition of 0.1 M Na_2_CO_3_ and the liberation of *p*-NP was detected continuously at 405 nm. One unit (1 U) of enzymatic activity was defined as the amount of enzyme needed to release 1 μM *p*-NP from the substrate per minute in the standard assay. The EstSJ kinetic parameters, *K*_*m*_ (mM) and *K*_*cat*_ (S^–1^), were determined using various concentrations of *p*-NPC_2_ (0.3 to 2.8 mM) and were calculated using the non-linear regression method based on GraphPad Prism 5 software. The value of *K*_*cat*_ was calculated according to *K*_*cat*_ = *V*_*max*_/*E*_*t*_ (*E*_*t*_ means enzyme concentration in the standard assay).

### Biochemical characterization of EstSJ

The effects of pH were investigated by incubating purified EstSJ in three different buffers (50 mM): citrate-phosphate (pH 4.0–7.5), Tris–HCl (pH 7.0–9.5), and borax-sodium hydroxide (pH 9.5–10.0). The effects of temperature were determined by pre-incubating EstSJ in 50 mM Tris–HCl buffer, pH 8.0, at various temperatures (0–70°C, 10°C intervals). The pH stability was measured by incubating EstSJ in various pH values at 30°C for 1 h, and temperature stability was evaluated by incubating EstSJ in 50 mM Tris–HCl buffer (pH 8.0) at 25, 40, 45, and 50°C for specific time intervals, respectively. The residual activity of EstSJ was measured after each treatment, and the highest assayed enzymatic activity at each pH or temperature was set as 100%.

To investigate the influence of different metal ions (Ca^2+^, K^+^, Mn^2+^, Mg^2+^, Cu^2+^, Ni^2+^, Na^+^, Li^+^, Co^2+^, Fe^2+^, Zn^2+^, Fe^3+^, Al^3+^, Hg^2+^, and Ag^+^) and chemical reagents [Tween 80, urea, ethylene diamine tetraacetic acid (EDTA), dithiothreitol (DTT), SDS, and hexadecyl trimethyl ammonium bromide (CTAB)] on EstSJ, the reactions were conducted separately by incubating them at a final concentration of 1 mM with EstSJ in 50 mM Tris–HCl buffer (pH 8.0) at 30°C for 5 min. K-ACET acetic acid kit was used to determine the deacetylation activity of EstSJ according to the manufacturer’s instructions by measuring the acetic acid released from 7-ACA. Specifically, the reaction mixture containing 0.1 mL EstSJ (111.7 μg) and 7.5 mM 7-ACA was incubated at 25°C for 10 min. Every test was conducted in triplicate and no enzyme was added to the reaction mixture in the control reaction for correction of spontaneous hydrolysis. One unit of EstSJ was designated as the amount of enzyme required for catalyzing the release of 1 μM acetic acid per minute. Meanwhile, activities of EstSJ on cefotaxime acid/cephalothin sodium/cefuroxime acid/7-ACA (50 mM) or tert-butyl acetate/linalyl acetate/glyceryl tributyrate/glyceryl trioleate/terpinyl acetate (25 mM) were detected by adding 400 μg EstSJ or EstSJ mutants and 0.02% bromothymol blue in Na_2_HPO_4_–KH_2_PO_4_ buffer (50 mM, pH 7.3).

### Homologous modeling and molecular docking

The crystal structure of YXIM (PDB: 2O14) from *B. subtilis*, which shares 95.36% sequence similarity with EstSJ, was used as a template to build the 3D structure of EstSJ by an online SWISS-MODEL server (Waterhouse et al., 2018). The online SAVES Server was applied to check the quality of the modeled structure.^2^ The chemical structure of 7-ACA was obtained from PubChem^3^ and the interaction between EstSJ and 7-ACA was predicted by AutoDock 4.2 (Morris et al., 2009). For the docking assays, polar hydrogen atoms were added both to the target protein and the ligand. In preparation, the docking poses were restricted to a grid that was of the dimension of 42 Å by 56 Å by 50 Å and centered on all-atom centers of three active-site residues (Ser^186^-Asp^354^-His^357^). All the obtained docking results and the EstSJ modeled structure were analyzed by PyMOL.

### Product detection

For the microTOF-Q II analysis, the reaction mixture with a total volume of 400 μL containing 0.1 mL (111.7 μg) of purified EstSJ and 15 mM 7-ACA in 50 mM Na_2_HPO_4_–KH_2_PO_4_ buffer, pH 7.3, was incubated at 25°C for 15 min, following termination by 5 mM H_2_SO_4_. D-7-ACA was identified by obtaining the ion *m/z* 253 corresponding to the D-7-ACA-Na adduct (Na monoisotopic weight 23). The system equipped with an electrospray ionization source (ESI) was operated under positive mode using the following conditions: nebulizer: 2.0 bar, dry gas 4.0 L min^–1^ at 200°C, and capillary set at 4,500 V–500 V. The analysis was recorded at 50–1,000 *m/z*.

## Results and discussion

### Sequence analyses

Based on the analysis of the genomic sequence of *B. subtilis* KATMIRA1933 (GenBank: JMEF00000000.1), a putative acetylesterase (Protein ID: KDE25464.1), named here EstSJ that has not been characterized yet, was annotated. The gene *estSJ* is 1,146 bp in length and encodes a protein of 382 amino acids. Its theoretical molecular weight and isoelectric point were 41,823.33 Da and 6.34, respectively. BLASTP showed that EstSJ belongs to the rhamnogalacturonan_acetylesterase-like SGNH hydrolase superfamily and displayed the highest similarity with YXIM (PDB: 2O14), an esterase with unknown function from *B. subtilis*. Multiple amino acid sequence alignments of EstSJ with two characterized SGNH hydrolase superfamily members, TAP-thioesterase I (PDB ID: 1IVN) from *E. coli* (Lo et al., 2003) and RGAE-rhamnogalacturonan acetylesterase (PDB ID: 1DEO) from *Aspergillus aculeatus* (Mølgaard et al., 2000), revealed that there was a GDSL motif but not GXSXG at the N-terminal of EstSJ and four conserved residues, Ser, Gly, Asn, and His (SGNH), located in blocks I, II, III, and V (Jones et al., 2020; Maršavelski et al., 2020; Supplementary Figure 1). Together with the putative catalytic triads (Ser^186^-Asp^354^-His^357^), the existence of conserved SGNH blocks suggested that they might also play important functions and stay conservative during evolution.

**FIGURE 1:**
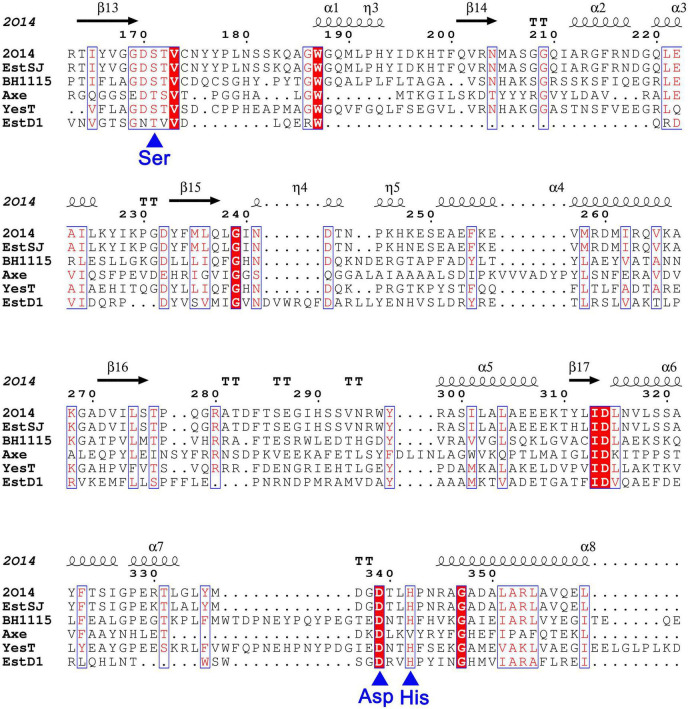
The sequence alignment of EstSJ with other characterized homologs using MEGA 7.0 and rendered by ESPript 3.0. EstSJ (KDE25464.1) from *B. subtilis* KATMIRA1933; YXIM (PDB ID: 2O14) from *B. subtilis*; BH1115 (BAB04834.1) from *B. halodurans* C-125; Axe (CAB76451.2) from *B. pumilus* PS213; YesT (O31523.1) from *B. subtilis* ATCC6633; and EstD1 (AIY63728.1) from *Alicyclobacillus tengchongensis*. Residues in white font shaded in red represent the ones strictly conserved between EstSJ and its homologs. Blue asterisks below the alignment represent the putative catalytic triad [Ser (S), Asp (D), and His (H)].

**FIGURE 2:**
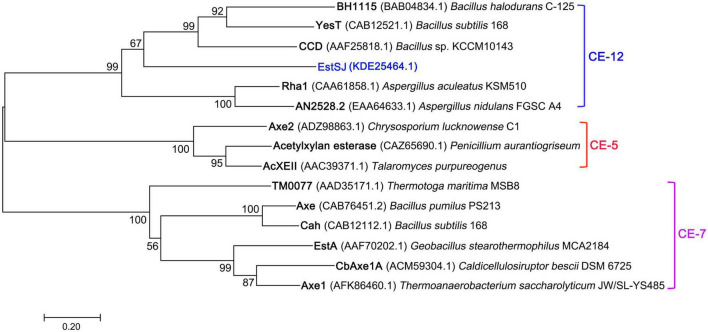
The phylogenetic tree of EstSJ and other characterized acetyl xylan esterases using the neighbor-joining method. A bootstrap analysis of 1,000 replicates was conducted, and values > 50% are shown. Sequences are retrieved from GenBank and their sources together with accession numbers are given.

In addition, four previously reported esterases with 7-ACA deacetylase activity were also aligned with EstSJ. EstSJ shared approximately 26.5, 37.04, 28.64, and 18.35% identities with BH1115 (BAB04834.1, CE12 family) (Navarro-Fernández et al., 2008), Axe (CAB76451.2, CE7 family) (Degrassi et al., 2000), YesT (CAB12521.1, CE12 family) (Martínez-Martínez et al., 2008), and EstD1 (AIY63728.1, CE12 family) (Ding et al., 2016), respectively (Figure 1). No sequence identity was found between EstSJ and other characterized 7-ACA deacetylases, such as TM0077 (AAD35171.1, CE7 family) (Levisson et al., 2012), AXE (AGF25253.1, CE7 family) (Tian et al., 2014), PbAcE (PDB ID: 6AGQ) (Park et al., 2018), and Axe1 (PDB ID: 3FCY, CE7 family) (Lorenz and Wiegel, 1997). A phylogenetic tree of EstSJ with these reported esterases was built using MEGA 7.0, and the results revealed that EstSJ clustered together with BH1115 and Yest in the CE12 family and formed an independent clade (Figure 2). Therefore, EstSJ represents a new member of the CE12 family that might show 7-ACA deacetylase activity and deserves to be further explored and characterized.

### Heterologous overexpression and purification

**FIGURE 3:**
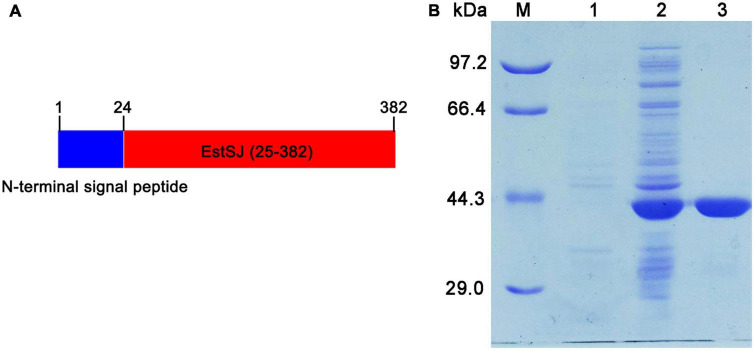
Purification of EstSJ. **(A)** Schematic diagram of the EstSJ amino acid sequence. In total, 1–24 amino acids represent the N-terminal signal peptides and 25–382 amino acids are chosen for EstSJ overexpression. **(B)** The purification of EstSJ. M, protein marker (kDa); 1, cell lysates of *E. coli* BL21(DE3) cells harboring the pACYCDuet-1 plasmid (control); 2, supernatant of *E. coli* BL21(DE3) cells containing recombinant plasmid pACYCDuet-1/EstSJ; and 3, purified EstSJ.

A signal peptide (1–24 amino acid residues) was predicted by SignalP 4.0 at the N-terminal of EstSJ, which might hamper its intracellular accumulation. Therefore, EstSJ (25–382 amino acid residues) with N-terminal truncation (1–24 amino acid residues) (Figure 3A) was inserted into the pACYCDuet-1 plasmid following transformation into *E. coli* BL21(DE3) competent cells for EstSJ heterologous overexpression. As shown in Figure 3B, the purified EstSJ appeared as a single band on the SDS-PAGE, which was closely consistent with its predicted theoretical molecular weight (39.1 kDa). The protein size of EstSJ is similar to those of other previously reported acetyl xylan esterases, such as TM0077 (37 kDa) (Levisson et al., 2012), EstZY (36.5 kDa) (Ding et al., 2020), and AXE (35.607 kDa) (Tian et al., 2014), which also displayed deacetylation activity on 7-ACA.

### Characterization of EstSJ

**FIGURE 4:**
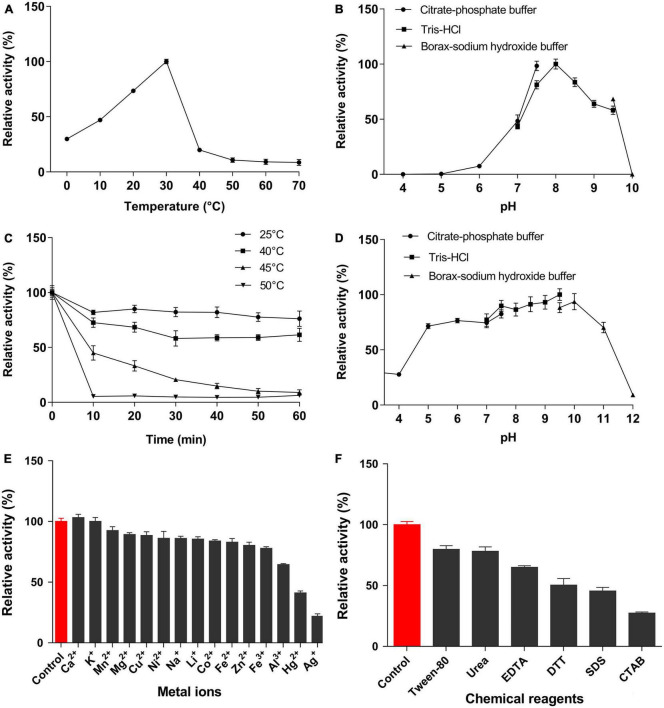
Characterization of EstSJ. Effects of **(A)** temperature, **(B)** pH, **(C)** temperature stability, **(D)** pH stability, **(E)** metal ions, and **(F)** chemical reagents on the EstSJ activity against *p*-NPC_2_. The results shown are means ± standard deviation.

EstSJ displayed the strongest preference toward *p*-NPC_2_, which is similar to AXE (Tian et al., 2014), EstZY (Ding et al., 2020), and TM0077 (Levisson et al., 2012), and its activity decreased sharply with the increase in the *p*-NP ester chain length (Supplementary Figure 2). The activity of EstSJ was investigated in different pH buffers (pH 4.0–10.0) and temperatures (0–70°C) using *p*-NPC_2_ as the substrate. Maximal activity of EstSJ was detected at pH 8.0 in Tris–HCl buffer, and >50% of its original activity was maintained across a pH range of 7.5–9.5. No activity was detected at both pH values of 5.0 and 10.0 (Figure 4B). EstSJ was stable at a pH range between 5.0 and 10.0, and it maintained 70∼90% of its original activity when incubated at these pH ranges for 1 h (Figure 4D), indicating that it might be applied under acidic or alkaline conditions. Among other previously characterized esterases that were active on 7-ACA, EstSJ was comparable to those of EstZY and AXE, which was stable between pH 7.0–11.0 (Ding et al., 2020) and pH 6.0–11.0 (Tian et al., 2014), respectively. To investigate the optimum temperature of EstSJ, the effects of various temperatures (0–70°C) on EstSJ were assayed. As shown in Figure 4A, the highest activity of EstSJ was observed at 30°C, which was similar to those of AcXEs (30°C), an esterase identified from a hot desert hypolith metagenomic DNA (Adesioye et al., 2018), but lower than that of EstA (95°C) (Levisson et al., 2009) from *T. maritima*, EstD1 (65°C) (Ding et al., 2016), or AXE (40°C) (Tian et al., 2014), respectively. The temperature stability was assessed by incubating EstSJ at 25, 40, 45, and 50°C for 1 h in 50 mM Tris–HCl buffer (pH 8.0), respectively, and the residual activities were determined. EstSJ maintained more than 70 and 50% of its original activity after incubation at 25°C and 40°C for 1 h, respectively, whereas the activity decreased sharply at 45 or 50°C and was almost completely lost in 10 min at 50°C (Figure 4C). Among the reported deacetylases, EstSJ may be more appropriate for 7-ACA deacetylation in practice because 7-ACA is unstable at a high temperature. Under optimum pH and temperature, the *K*_*m*_, *K_*cat*_/K_*m*_*, and specific activity of EstSJ toward *p*-NPC_2_ were 3.03 ± 0.40 mM, 4652.69 S^–1^ mM^–1^, and 1783.52 U mg^–1^, respectively (Supplementary Table 2). Previous reports showed that the enzymatic activities of AXE (Tian et al., 2014), EstZY (Ding et al., 2020), and TM0077 (Levisson et al., 2012) toward *p*-NPC_2_ were 2,949, 221.25, and 113.5 ± 1.5 U mg^–1^, respectively.

The effects of metal ions or chemical reagents on EstSJ were investigated. As shown in Figure 4E, Ca^2+^ increased the activity of EstSJ slightly (3% activation), while Mn^2+^, Mg^2+^, Cu^2+^, Ni^2+^, Na^+^, Li^+^, Co^2+^, Fe^2+^, Fe^3+^, and Zn^2+^ exhibited an 8∼23% inhibition on EstSJ. Al^3+^ had moderate inhibitory effects (36% inhibition), and K^+^ had no obvious influence on EstSJ. Hg^2+^ and Ag^+^ displayed 59 and 79% inhibition on EstSJ, respectively. For the chemical reagents, Tween 80 [1% (v/v)], urea (1 mM), and EDTA (1 mM) inhibited EstSJ moderately by 21, 22, and 35%, respectively. DTT, SDS, and CTAB inhibited EstSJ strongly by 50, 55, and 73% (Figure 4F). Similar to AXE, Ca^2+^ also increased the activity of AXE by 2% activation. Fe^3+^, Cu^2+^, and Zn^2+^ also inhibited AXE, but with 86, 52, and 56% inhibition, respectively, higher than that of EstSJ (Tian et al., 2014). Collectively, the good tolerance of EstSJ to metal ions or chemical reagents suggested that it could be a promising candidate in practical pharmaceutical use.

### Homology and molecular docking

**FIGURE 5:**
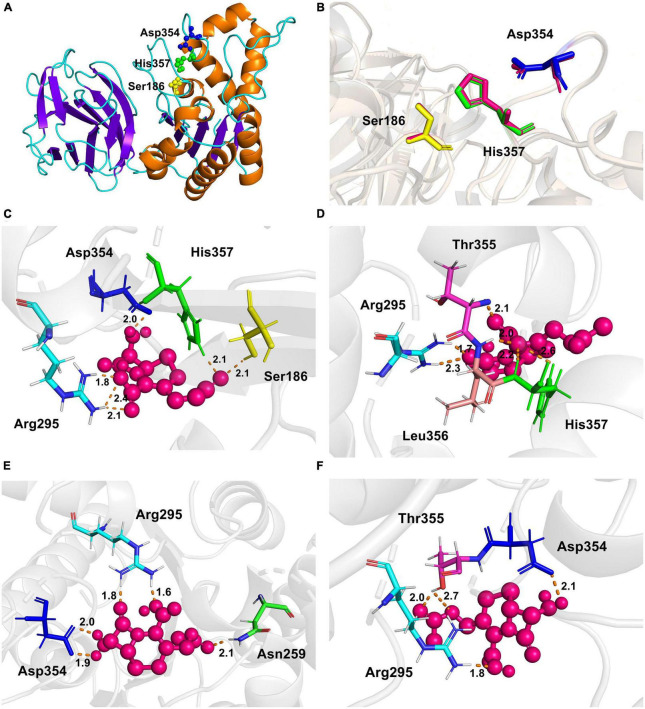
The overall structure of EstSJ. **(A)** The modeled structure of EstSJ using YXIM (PDB: 2O14) from *B. subtilis*, which exhibited 95.36% identity with EstSJ as a template. **(B)** A structural comparison of the modeled EstSJ and YXIM, and the catalytic residues are shown in stick. **(C–F)** Interactions between active sites (Ser^186^, Asp^354^, and His^357^) and other residues (Asn^259^, Arg^295^, Thr^355^, and Leu^356^) with 7-ACA. 7-ACA and hydrogen bonds are indicated by pink ball and dash orange lines.

The experimentally determined protein structures provided a bridge to study the structure unknown proteins through homology modeling, such as to identify the putative catalytic or specific substrate-binding residues that are important for enzyme activity (Waterhouse et al., 2018). The crystal structure of YXIM (PDB: 2O14) from *B. subtilis* sharing 95.36% similarity with EstSJ was employed as a template to create the homologous 3D structural model of EstSJ. The first 24 amino acid residues were not modeled. To date, a functional study about YXIM toward 7-ACA or cephalosporin C has not been observed yet. As shown in Figure 5A, the overall structure of EstSJ is represented as having two domains, out of which one domain contains the catalytic triad (Ser^186^-Asp^354^-His^357^), which is similar to BH1115, a rhamnogalacturonan acetyl esterase, which also belongs to the CE12 family from *B. halodurans* (Navarro-Fernández et al., 2008). This domain was composed of five β-strands surrounded by α-helices in an αβα sandwich fold. At the same time, SAVES v6.0 was used to evaluate the model. The Ramachandran plot shows that 92.5% of amino acids fall in the most favorable area (Supplementary Figure 3), indicating that the modeled EstSJ was of acceptable quality. Structural comparisons of modeled EstSJ and YXIM also revealed that their catalytic triads (Ser^186^-Asp^354^-His^357^) matched together (Figure 5B). To verify whether Ser^186^-Asp^354^-His^357^ was composed of the catalytic triad of EstSJ, *E. coli* BL21(DE3) competent cells containing pACYCDuet-1/EstSJ^*Ser*186*A*^, pACYCDuet-1/EstSJ^*Asp*354*A*^, and pACYCDuet-1/EstSJ^*His*357*A*^ were separately constructed, and the EstSJ^*Ser*186*A*^, EstSJ^*Asp*354*A*^, and EstSJ^*His*357*A*^ mutants were heterologously overproduced and purified (Supplementary Figure 4A). As shown in Supplementary Table 2, EstSJ lost almost all of its original activity after Ser^186^, Asp^354^, and His^357^ were substituted with A, respectively, indicating that Ser^186^-Asp^354^-His^357^ composed the catalytic triad of EstSJ.

### Functional analysis of EstSJ

**FIGURE 6:**
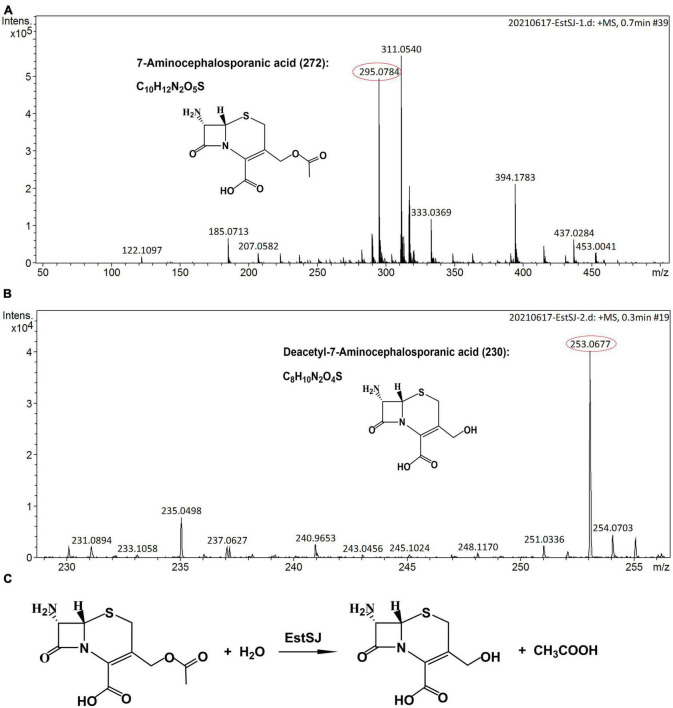
The microTOF-Q II analysis. **(A)** 7-ACA (15 mM) incubated in 50 mM Na_2_HPO_4_–KH_2_PO_4_ buffer, pH 7.3, at 25°C for 15 min without EstSJ (control). **(B)** 7-ACA (15 mM) incubated in 50 mM Na_2_HPO_4_-KH_2_PO_4_ buffer, pH 7.3, at 25°C for 15 min with 0.1 mL EstSJ (111.7 μg). **(C)** 7-ACA deacetylation by EstSJ.

Deacetylation of cephalosporins and other important esters, such as tertiary acetate esters, catalyzed by EstSJ and its three mutants (EstSJ^*Ser*186*A*^, EstSJ^*Asp*354*A*^, and EstSJ^*His*357*A*^) was determined. In the pharmaceutical chemistry, tertiary alcohols, which are mainly obtained through the enzymatic hydrolysis of tertiary acetate esters, are critical building blocks. Therefore, activities of EstSJ toward tert-butyl acetate, linalyl acetate, glyceryl tributyrate, glyceryl trioleate, and terpinyl acetate were investigated. The release of acetic acid could be monitored by observing a color change from blue to yellow using a pH indicator-based colorimetric assay. Unfortunately, no color change was monitored, indicating that EstSJ displayed no activity on the above esters (data not shown). As shown in Supplementary Figure 5, EstSJ was active on 7-ACA, cefotaxime acid, and cephalothin sodium and no activity was detected on cefuroxime acid. The microTOF-Q II analysis was used to further characterize the deacetylating product of 7-ACA by purified EstSJ. As shown in Figure 6, a peak with *m/z* 295.0784 [M + Na]^+^ in the control without EstSJ (Figure 6A), and the deacetylated derivative D-7-ACA with *m*/*z* 253.0677 [M + Na]^+^ in the reaction with EstSJ were detected (Figure 6B). In addition, the deacetylase activity of EstSJ toward 7-ACA was determined as 4.50 U mg^–1^, with *K*_*m*_ and *K*_*cat*_/*K*_*m*_ values being 25.66 ± 5.55 mM and 2.98 S^–1^ mM^–1^, respectively. Meanwhile, no obvious activity was detected for EstSJ^*Ser*186*A*^ and EstSJ^*His*357*A*^, and the activity of EstSJ^*Asp*354*A*^ on 7-ACA was 1.03 U mg^–1^, which also supports the result observed in Supplementary Figure 5. Combined with all the results mentioned above, a reaction mode of 7-ACA catalyzed by EstSJ was proposed (Figure 6Cx).

To better elucidate the binding and catalytic mechanisms of EstSJ on substrate 7-ACA, a molecular docking analysis of the receptor (EstSJ) and ligand (7-ACA) was performed. The results showed that the ligand 7-ACA was located, beside the active residues Ser^186^, Asp^354^, and His^357^, in the cavity (Figure 5C) and interacted with other amino acid residues, namely, Asn^259^, Arg^295^, Thr^355^, and Leu^356^ (Figures 5C–F). To investigate their functions, *E. coli* BL21(DE3) cells containing pACYCDuet-1/EstSJ^Asn259A^, pACYCDuet-1/EstSJ^Arg295A^, pACY CDuet-1/EstSJ^Thr355A^, and pACYCDuet-1/EstSJ^Leu356A^ plasmids were constructed separately, and the N-terminal His_6_-tagged EstSJ^Asn259A^, EstSJ^Arg295A^, EstSJ^Thr355A^, and EstSJ^Leu356A^ mutants were heterologously overproduced and purified (Supplementary Figure 4B), following which the activities and kinetic parameters on 7-ACA were determined. As shown in Table 1, activities of EstSJ^Asn259A^, EstSJ^Arg295A^, EstSJ^Thr355A^, and EstSJ^Leu356A^ mutants were approximately 1.44, 1.06, 1.66, and 0.63 U mg^–1^, which were about 32, 23.6, 36.9, and 14% of the original activity of the wild-type EstSJ. These results suggested that these amino acid residues might be involved in 7-ACA binding. Although 7-ACA is a crucial intermediate for synthesizing industrial cephalosporin antibiotics, its continuous production and uncontrolled use have led to their widespread presence in aquatic environments (Yu et al., 2017). More residues have been detected in animal tissues, treated sewage, surface water bodies, or hospital effluents which have caused immediate or potential threats to human and environmental safety (Chen et al., 2017). Currently, various methods, including chemical oxidation processes and biological treatments, are being developed to remove 7-ACA from wastewater. However, the products are usually more toxic than their parent compounds after being treated by chemical oxidation processes. On the other hand, the biological methods, especially bacterial communities, caused a significant impact on the removal of 7-ACA, and enzymatic activities of hydrolysis were environment friendly and cost-effective (Hu et al., 2020). Therefore, functional 7-ACA acetylesterases, including EstSJ, might also be used for environmental 7-ACA bioremediation.

**TABLE 1:** Kinetic parameters of EstSJ and its mutants on 7-ACA*^a^*.

Enzyme	*K*_*m*_ (mM)	*V*_*max*_ (mM min^–^^1^ mg^–^^1^)	*K*_*cat*_ (S^–1^)	*K_*cat*_/K_*m*_* (S^–^^1^ mM^–^^1^)	Specific activity (U mg^–^^1^)
EstSJ	25.66 ± 5.55	117.10 ± 12.52	76.54 ± 12.52	2.98 ± 0.15	4.50
EstSJ^Asn259A^	172.10 ± 59.09	167.00 ± 48.37	108.72 ± 48.37	0.63 ± 0.06	1.44
EstSJ^Arg295A^	40.62 ± 10.14	54.64 ± 8.44	35.71 ± 8.44	0.88 ± 0.01	1.06
EstSJ^Thr355A^	16.30 ± 1.99	33.28 ± 1.62	21.75 ± 1.62	1.33 ± 0.06	1.66
EstSJ^Leu356A^	47.27 ± 9.12	31.65 ± 3.92	20.68 ± 3.92	0.43 ± 0.01	0.63

^a^Reactions were conducted in triplicate in 50 mM Na_2_HPO_4_–KH_2_PO_4_ buffer, pH 7.3, at 30°C in the standard assay.

## Conclusion

In this study, an acetylesterase, EstSJ, a new member of the CE12 family, was identified and characterized. EstSJ prefers short-chain acyl esters from *p*-NPC_2_ to *p*-NPC_6_ and acquires its highest activity at 30°C and pH 8.0. EstSJ can remove the 3′ acetyl group from 7-ACA to generate deacetylated 7-ACA, which is a critical intermediate in the industry for synthesizing new types of β-lactam antibiotics. Meanwhile, EstSJ is an SGNH family esterase with a typical N-terminal GDS(X) motif and this study will also broaden our general understanding of esterases from the SGNH superfamily. In future, protein engineering technologies, namely, directed evolution and protein rational/semirational design, might be used together to improve the deacetylation activity of esterases against 7-ACA.

## Data availability statement

The datasets presented in this study can be found in online repositories. The names of the repository/repositories and accession number(s) can be found in the article/ Supplementary material.

## Author contributions

XW and SN performed the experiments and prepared the original draft. JL analyzed the data. QW and ZH provided the resources. JD and BX conceived, designed the experiments, and edited the manuscript. JD supervised the study. All authors read and approved the final manuscript.

## Footnotes

1
http://www.cazy.org/

2
https://saves.mbi.ucla.edu/

3
https://pubchem.ncbi.nlm.nih.gov/
