# Supplementary material for: Biochemical characterization of an acetylesterase from Bacillus subtilis and its application for 7-aminocephalosporanic acid deacetylation
Source: Front Microbiol. 2023 May 3;14:1164815. doi: 10.3389/fmicb.2023.1164815 (PMC10189120; doi:10.3389/fmicb.2023.1164815)
Supplement: Supplementary file 1 [file Data_Sheet_1.pdf]

Supplementary Material

Biochemical Characterization of an Acetylesterase From *Bacillus subtilis* and Its Application for 7-Aminocephalosporanic Acid Deacetylation

Xiaoliang Wang[1†], Sujin Nong[1†], Jiayi Li[1], Yan Liu[1], Qian Wu[1], Zunxi Huang[1], Bo Xu[1*], Junmei Ding[1*]

[1]Engineering Research Center of Sustainable Development and Utilization of Biomass Energy, Ministry of Education, Yunnan Normal University, Kunming, China

*** Correspondence:** Bo Xu: xubo@ynnu.edu.cn; Junmei Ding: djm@ynnu.edu.cn (J. Ding).

[†]These authors contributed equally to this work and share first authorship.

Supplementary Figures and Tables

Supplementary Figures

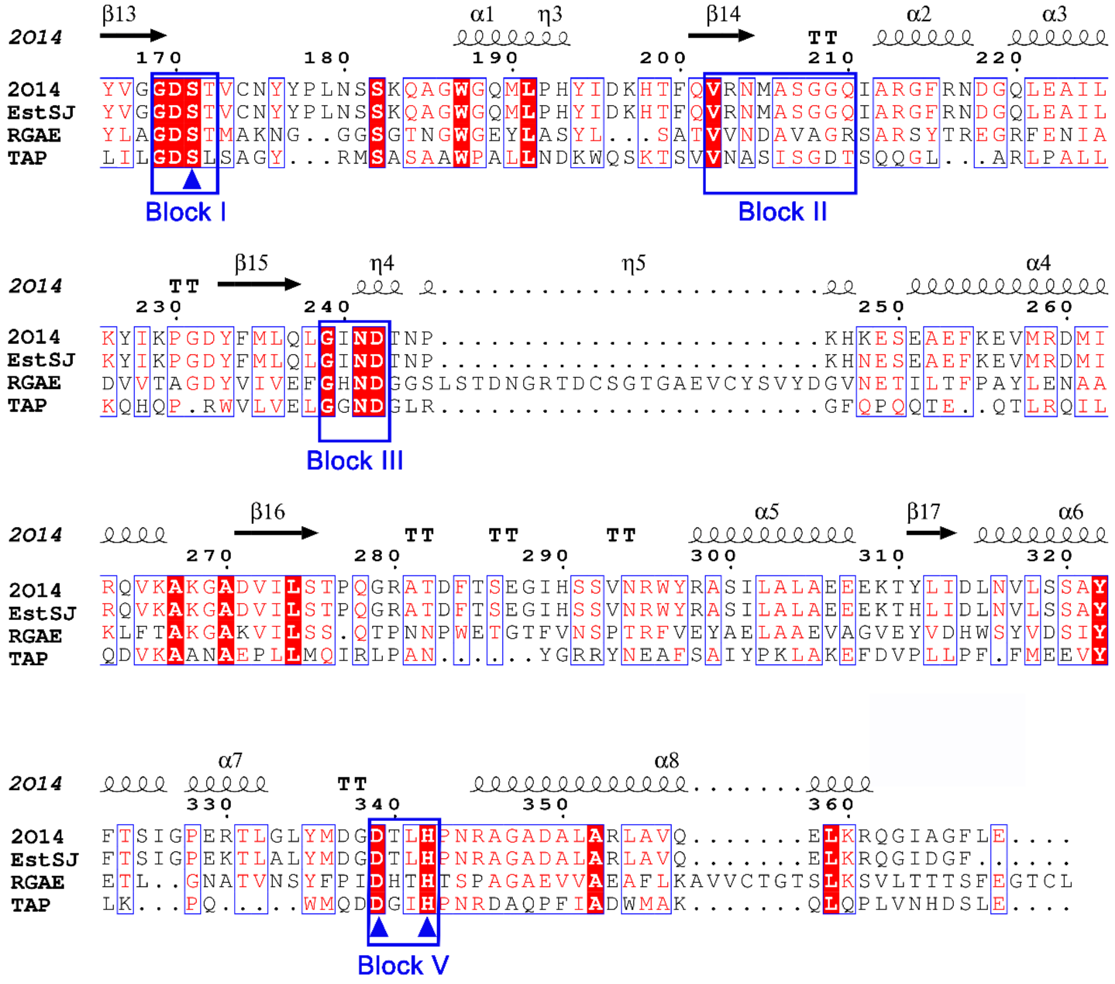

Supplementary Figure 1. Multiple amino acid sequence alignment of EstSJ with other characterized members of SGNH-hydrolase family. EstSJ (KDE25464.1) from *B. subtilis* KATMIRA1933; YXIM (PDB ID: 2O14) from *B. subtilis*; TAP-thioesterase I (PDB ID: 1IVN) from *E. coli*; RGAE-rhamnogalacturonan acetylesterase (PDB ID: 1DEO) from *Aspergillus aculeatus*. MEGA 7.0 was used to align the sequences and ESPript 3.0 was used to render the results. Residues strictly conserved among groups are shown in white font on a red background. Four conserved sequence blocks (Block I-V) found in all SGNH hydrolases are boxed. Block I have the characteristic GDS sequence motif; Block II has a Gly as the only conserved residue in the members of this family; GXND is the consensus sequence in block III; Finally, block V has the DXXHP conserved sequence. The symbols above the sequences represent the secondary structure. Blue triangles indicate the catalytic triad.

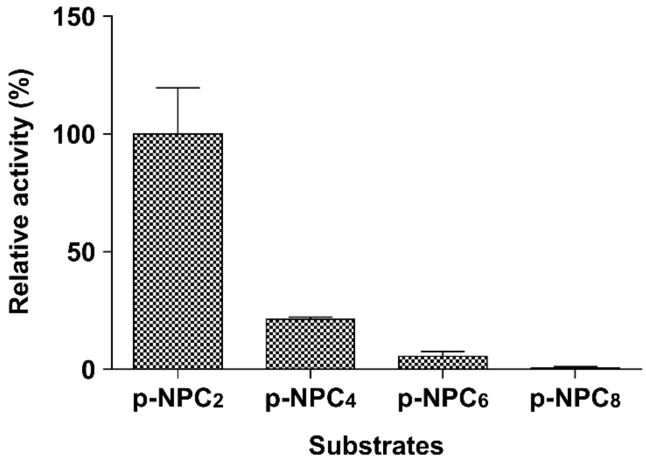

Supplementary Figure 2. Substrate specificity of EstSJ. Reactions were performed using standard assay with various length of *p*-NP esters as substrates at 30°C, pH 8.0.

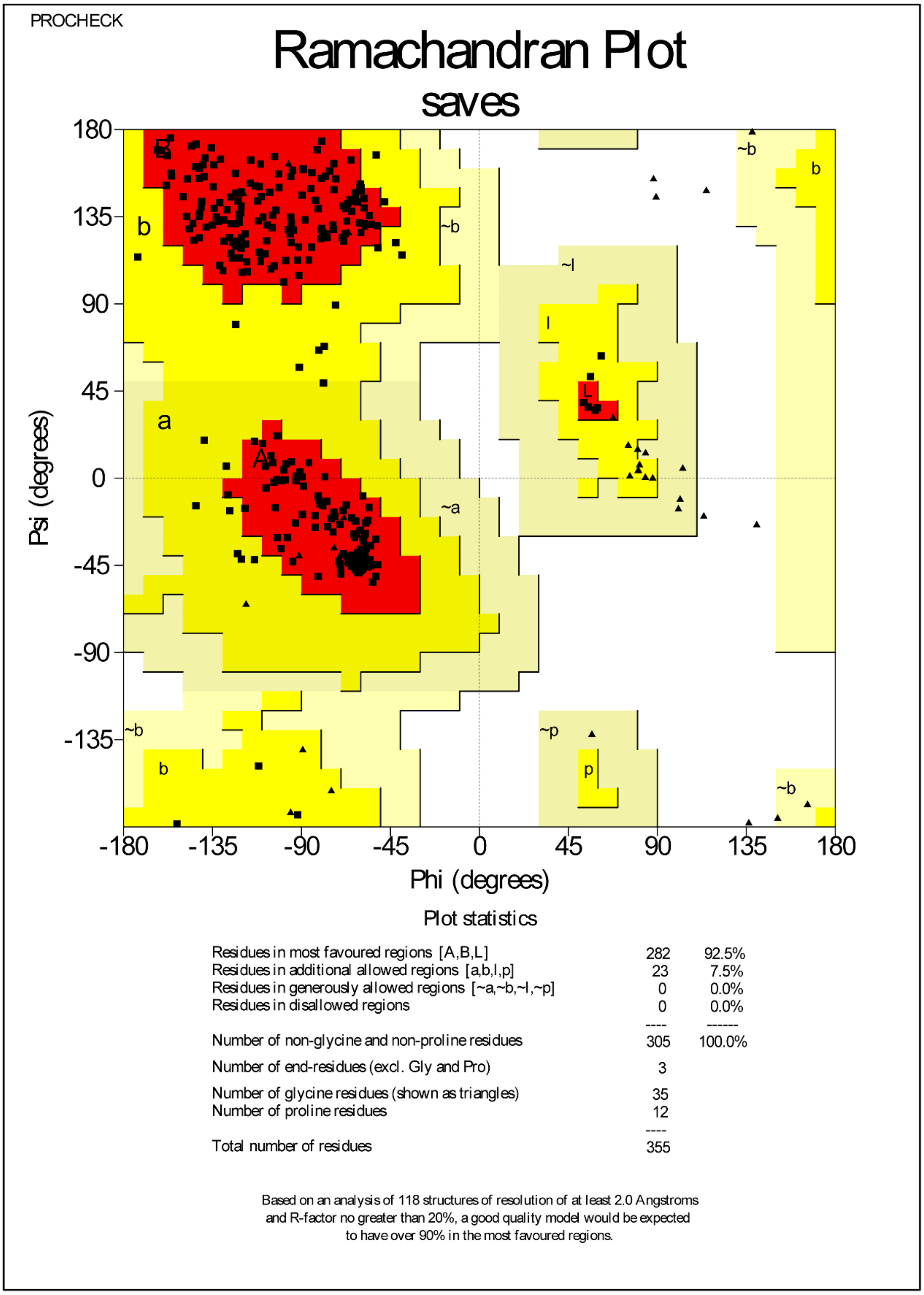

Supplementary Figure 3. Ramachandran plot of modeled EstSJ. There are 92.5% amino acid residues located in most favored regions, indicating that the predicted 3D structure of EstSJ is reliable and acceptable.

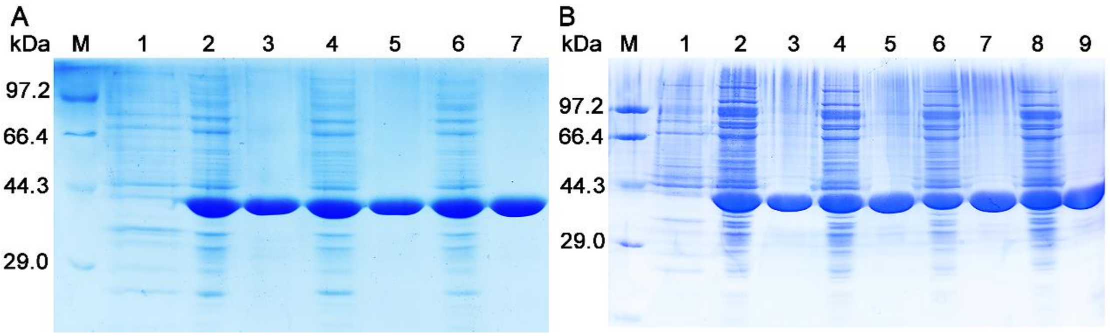

Supplementary Figure 4. Site-directed mutagenesis of EstSJ. **(A)** Purification of EstSJ^{Ser186A}, EstSJ^{Asp354A} and EstSJ^{His357A} mutants. Lane M: protein marker (kDa); Lane 1: cell lysates of *E. coli* BL21(DE3) cells harboring pACYCDute-1 plasmid (control); Lane 2, 4, 6: cell lysates of *E. coli* BL21(DE3) cells carrying pACYCDute-1/EstSJ^{Ser186A}, pACYCDute-1/EstSJ^{Asp354A}, and pACYCDute-1/EstSJ^{His357A} recombinant plasmids, respectively; Lane 3, 5, 7: purified EstSJ^{Ser186A}, EstSJ^{Asp354A} and EstSJ^{His357A} mutants. **(B)** Purification of EstSJ^{Asn259A} (Lane 3), EstSJ^{Arg295A} (Lane 5), EstSJ^{Thr355A} (Lane 7), and EstSJ^{Leu356A} (Lane 9) mutants. Lane 2, 4, 6, and 8: cell lysates of *E. coli* BL21(DE3) cells carrying pACYCDute-1/EstSJ^{Asn259A}, pACYCDute-1/EstSJ^{Arg295A}, pACYCDute-1/EstSJ^{Thr355A}, and pACYCDute-1/EstSJ^{Leu356A} recombinant plasmids, repectively.

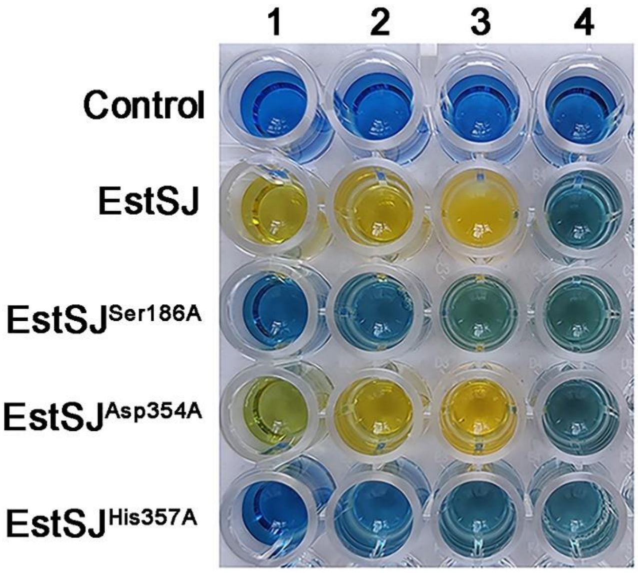

Supplementary Figure 5. Deacetylation activities of EstSJ. Hydrolysis of cephalosporins by EstSJ and its mutants. 1. 7-ACA; 2. cefotaxime acid; 3. cephalothin sodium; 4. cefuroxime acid.

Supplementary Table 1. Primers used for site-directed mutagenesis in this study.

EstSJ^{Ser186A}	Forward	5'-ATTTATGTCGGCGGCGACGCGACGGTGTGCA-3'
	Reverse	5'-CGTCGCCGCCGACATAAATGGTTCGGTTCGT-3'
EstSJ^{Asp354A}	Forward	5'-CTTTATATGGACGGAGCTACGCTTCAT-3'
	Reverse	5'-GCTCCGTCCATATAAAGCGCGAGTGT-3'
EstSJ^{His357A}	Forward	5'-GAGATACGCTTGCTCCGAACCG -3'
	Reverse	5'-GCAAGCGTAT CTCCGTCCAT ATAAAGC-3'
EstSJ^{Asn259A}	Forward	5'-TCGGCATTAATGACACAGCTCCGAAGCAT-3'
	Reverse	5'- TGTGTCATTA ATGCCGAGCT GCAACATA -3'
EstSJ^{Arg295A}	Forward	5'- TATCAACGCCCCAGGGCGCTGCGACCGATT-3'
	Reverse	5'- GCCCTGGGGCGTTGATAGGATAACGTC-3'
EstSJ^{Thr355A}	Forward	5'-CTTTATATGGACGGAGATGCGCTTCATCC-3'
	Reverse	5'-ATCTCCGTCC ATATAAAGCGCGAGTGT-3'
EstSJ^{Leu356A}	Forward	5'-CTTTATATGGACGGAGATACGGCTCATCCGAAC-3'
	Reverse	5'-CGTATCTCCGTCCATATAAAGCGCGA-3'

The modified codons are underlined.

Supplementary Table 2. Kinetic parameters of recombinant EstSJ and its variants.

**Enzyme**	K_m **(mM)**	V_{max} **(mM min^{-1} mg^{-1})**	K_{cat} **(S^{-1})**	K_{cat}/K_m **(S^{-1} mM^{-1})**	**Specific activity (U mg^{-1})**
EstSJ	3.03 ± 0.40	21.64 ± 1.89	14102.31 ± 1.89	4652.69 ± 613.79	1783.52
S186A	4.80 ± 1.40	0.08 ± 0.02	49.48 ± 0.02	10.29 ± 2.96	2.38
D354A	12.69 ± 4.65	2.61 ± 0.84	1700.88 ± 0.84	134.03 ± 49.04	53.44
H357A	8.02 ± 3.06	0.45 ± 1.42	295.93 ± 1.42	36.89 ± 13.90	11.05

Reactions were conducted in triplicates in 50 mM Tris-HCl (pH 8.0), using *p*-NPC_2 (0.3 to 2.8 mM) as substrate at 30°C under standard assay.
